# A Rare Case of Vaginal Atresia in an Adolescent Girl Presenting With Abdominal Pain

**DOI:** 10.7759/cureus.46571

**Published:** 2023-10-06

**Authors:** Shahad Alotaibi, Ola Alotaibi, Roweim Sharaf, Raja Qaheri, Rania Alshamrani

**Affiliations:** 1 Medicine, Sulaiman Alrajhi University, Al Bukairiyah, SAU; 2 Pediatrics, King Fahad Medical City, Riyadh, SAU; 3 General Practice, King Abdulaziz University, Jeddah, SAU; 4 General Practice, First Moscow State Medical University, Moscow, RUS; 5 Pediatrics, Dallah Hospital, Riyadh, SAU

**Keywords:** case report, magnetic resonance imaging, acute abdomen, vaginal atresia, hematometra

## Abstract

Vaginal atresia, an infrequent congenital anomaly characterized by the absence or underdevelopment of the vaginal canal, presents significant complexities in pediatric and adolescent gynecological practice. Its diverse range of clinical presentations, including primary amenorrhea and cyclic abdominal discomfort, creates diagnostic challenges, highlighting the need for timely intervention to relieve symptoms and preserve future reproductive health. This case underscores the essential role of a collaborative, multidisciplinary approach involving pediatricians, gynecologists, and surgeons in ensuring comprehensive care and optimizing patient outcomes. In this report, we present the case of a 10-year-old female who initially presented with chronic abdominal pain, which had been occurring intermittently over the course of several months. This ultimately led to the diagnosis of hematometra secondary to vaginal atresia. Utilizing magnetic resonance imaging, we confirmed the diagnosis without exposing the patient to radiation, prioritizing her safety. A successful surgery was performed to create a working vaginal canal. The patient received careful postoperative care, and we closely followed her progress to support a smooth recovery.

## Introduction

Vaginal atresia, a congenital anomaly characterized by the absence or underdevelopment of the vaginal canal, is a rare and often challenging condition encountered in pediatric and adolescent gynecology [1]. It can present with a myriad of clinical symptoms, ranging from absent menses to cyclic abdominal pain, making the diagnosis a complex puzzle for healthcare providers [1]. While vaginal atresia is an uncommon finding, prompt and accurate diagnosis is crucial, as it not only addresses the immediate symptoms but also has profound implications for the patient's future reproductive and sexual health. Understanding the clinical presentation, diagnostic modalities, and management strategies for vaginal atresia is of paramount importance for healthcare providers, as timely intervention can alleviate symptoms and improve the quality of life for affected individuals [1,2]. Vaginal atresia, a congenital anomaly characterized by the absence or underdevelopment of the vaginal canal, is a rare and often challenging condition encountered in pediatric and adolescent gynecology. It can present with a myriad of clinical symptoms, ranging from absent menses to cyclic abdominal pain, making the diagnosis a complex puzzle for healthcare providers [2].

## Case presentation

A 10-year-old female child was admitted to our pediatric department with a chief complaint of acute abdominal pain. The patient was previously healthy with no significant medical history, and her parents reported that she had been experiencing intermittent abdominal pain over the past few months, which had progressively worsened over the last week. The pain had been occurring intermittently for approximately three months. The pain was localized to the lower abdomen and was associated with nausea and occasional vomiting. There were no associated urinary symptoms, fever, or recent history of trauma. Additionally, the patient was prepubescent, had not reached menarche, and had not experienced any prior abdominal or pelvic surgeries. Her abdominal pain had an insidious onset over the past few months and was characterized by cyclical episodes with no reported use of medications for symptom relief.

Upon initial evaluation, the patient appeared uncomfortable and was in moderate distress because of abdominal pain. No dysmorphic features were observed. Additionally, the patient was at Tanner Stage 1. Her vital signs were stable, with a heart rate of 100 beats per minute, blood pressure of 110/70 mm Hg, respiratory rate of 20 breaths per minute, and afebrile with a temperature of 37°C. Her weight and height were within the normal range for her age. Physical examination revealed tenderness in the lower abdomen, particularly in the suprapubic region, without any palpable masses or organomegaly. No signs of peritonitis were observed, and there were no signs of trauma, bruises, or any indicators of domestic or sexual abuse.

Given the nature and severity of the abdominal pain, a thorough work-up was initiated. Laboratory investigations, including a complete blood count and comprehensive metabolic panel, were within normal limits. Urinalysis showed no signs of infection or hematuria (Table 1).

**Table 1:** Initial Presentation Laboratory Values

Laboratory Parameter		Units of Measurement	Actual Value	Normal Reference Range
Complete Blood Count	Hemoglobin	g/dL	12.5	11.5-15.5
	White Blood Cell Count	cells/μL	7,200	4,000-11,000
	Platelet Count	cells/μL	250,000	150,000-450,000
Comprehensive Metabolic Panel	Sodium	mmol/L	138	135-145
	Potassium	mmol/L	4	3.5-5.1
	Creatinine	mg/dL	0.8	0.6-1.3
Urinalysis	White Blood Cells	cells/μL	3	<5 per high-power field
	Red Blood Cells	cells/μL	1	0-2 per high-power field

Considering the need for a more detailed assessment and the desire to minimize radiation exposure in this pediatric patient, a decision was made to opt for magnetic resonance imaging rather than a computed tomography scan. This choice was made to ensure the patient's safety and minimize radiation exposure, which is particularly important in pediatric cases.

**Figure 1:**
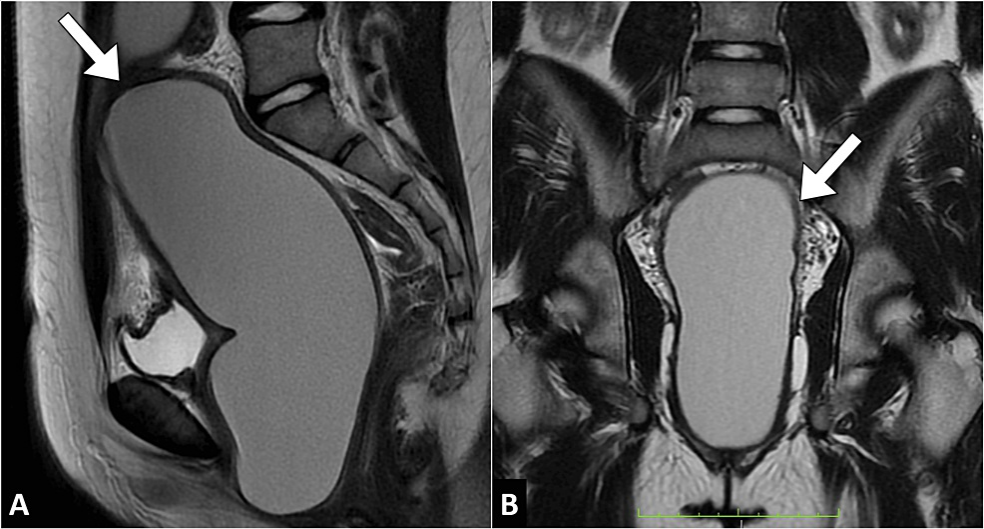
Sagittal (A) and coronal (B) T2-weighted MR images of the pelvis reveal an elongated and dilated uterus filled with blood products (arrow), consistent with hematometra. This condition exerts a notable mass effect in the pelvis, accompanied by the absence of the vagina. MR: magnetic resonance

The magnetic resonance imaging of the pelvis was subsequently performed, revealing a distended and elongated uterus filled with blood products. The uterus caused a significant mass effect in the pelvis. Furthermore, there was a conspicuous absence of the normal vaginal canal, replaced by a small amount of fibrous tissue. These findings were consistent with hematometrocolpos due to vaginal atresia (Figure 1).

With the diagnosis in hand, the patient's case was discussed with pediatric surgery and gynecology specialists. It was determined that surgical intervention would be necessary to address the vaginal atresia. The parents were informed about the condition and the proposed treatment plan, and they provided informed consent for the surgical procedure.

The patient underwent surgical correction of the vaginal atresia under general anesthesia. The surgery involved creating a vaginal canal to allow for normal menstrual flow and future sexual function. The procedure was successful, and the patient had an uneventful postoperative course. The surgical technique used was McIndoe vaginoplasty, and no complications, such as bleeding necessitating blood transfusion, occurred. A vaginal mold was placed after the surgery to maintain the neovagina and created a vaginal canal.

During her hospitalization, the patient received appropriate postoperative care, including pain management, wound care, and instructions for hygiene. A vaginal mold was placed and was an essential part of the long-term follow-up process.

## Discussion

Vaginal atresia, a rare congenital anomaly of the female reproductive system, poses unique diagnostic and management challenges. This comprehensive discussion delves into various aspects of vaginal atresia, including its clinical presentation, diagnosis, and treatment strategies, while emphasizing the importance of multidisciplinary care in achieving optimal outcomes for affected individuals [1,3].

Vaginal atresia is a condition wherein the urogenital sinus fails to contribute to the formation of the lower portion of the vagina, while the upper reproductive structures remain intact. This differs from vaginal agenesis, where the proximal portion of the vagina is absent. It is a rare condition, with an estimated incidence ranging from 1:4,000-1:10,000 live female births [1]. The rare prevalence of this condition often leads to its categorization within broader classifications in medical classification systems. For example, the American Society for Reproductive Medicine considers it a part of müllerian agenesis and dysgenesis [4].

Clinically, the most common presentation of vaginal atresia is primary amenorrhea in adolescence. However, some patients may present with abdominal pain during puberty, necessitating surgical drainage and vaginal reconstructions. While not typically associated, some individuals with vaginal atresia have reported lower urinary tract symptoms, possibly related to the proximity of bladder and urethra development to the vaginal plate [5].

Accurate diagnosis of vaginal atresia is crucial for appropriate management. The diagnostic process involves a comprehensive evaluation, which includes medical history, physical examination, imaging studies, hormonal testing, chromosomal analysis, and additional evaluations when necessary [3,6].

The treatment of vaginal atresia is primarily surgical, with the goal of creating a functional vaginal opening [3]. The specific approach depends on the severity of the condition and individual patient factors. Treatment options include vaginal dilation, vaginoplasty, tissue grafting, and tissue expanders. Medication alone does not treat vaginal atresia; however, hormonal therapy may be prescribed in some cases for temporary manipulation of the menstrual cycle until surgical correction is performed [6].

In recent years, there have been advancements in surgical techniques for vaginal reconstruction [7]. Robotic-assisted reconstruction using grafts such as small intestinal submucosa has shown promise in achieving successful outcomes with minimal complications [8]. However, it is important to note that the optimal surgical approach for vaginal atresia remains a topic of debate within the medical community. The choice of treatment should be individualized, taking into account the patient's specific condition and goals [2,7].

## Conclusions

In conclusion, this case underscores the significance of including gynecological conditions in the differential diagnosis of adolescent patients experiencing cyclic abdominal pain. It emphasizes the importance of considering congenital anomalies like vaginal atresia, particularly when there is a history of cyclic abdominal discomfort. Utilizing imaging studies for diagnosis proves to be a valuable tool in such cases. Timely diagnosis is of utmost importance, as it is pivotal for relieving the patient's symptoms promptly and ensuring their well-being.
